# Diagnostic yield of liquid‐based cytology in serial pancreatic juice aspiration cytological examination

**DOI:** 10.1002/deo2.177

**Published:** 2022-10-25

**Authors:** Koh Kitagawa, Akira Mitoro, Fumimasa Tomooka, Shohei Asada, Yukihisa Fujinaga, Norihisa Nishimura, Kosuke Kaji, Hideto Kawaratani, Takemi Akahane, Takahiro Ozutsumi, Miki Kaneko, Yuki Fujimoto, Yuki Tsuji, Masahide Enomoto, Soichi Takeda, Koji Murata, Takahiro Kubo, Satoshi Iwai, Aritoshi Koizumi, Akihiko Shibamoto, Junya Suzuki, Misako Tanaka, Takuya Matsuda, Nobuyuki Yorioka, Hiroyuki Masuda, Masayoshi Takami, Hitoshi Yoshiji

**Affiliations:** ^1^ Department of Gastroenterology Nara Medical University Nara Japan; ^2^ Division of Endoscopy Nara Medical University Nara Japan

**Keywords:** ENPD, ERCP, pancreatic cancer, pancreatic juice cytology, liquid‐based cytology

## Abstract

**Objectives:**

Serial pancreatic juice aspiration cytological examination (SPACE) via endoscopic retrograde cholangiopancreatography is a useful diagnostic method for early‐stage pancreatic cancer, such as carcinoma in situ that are difficult to diagnose by endoscopic ultrasound‐guided fine needle aspiration (EUS‐FNA). However, the diagnostic accuracy of SPACE is low, which is attributed to problems regarding specimen treatment. Hence, we evaluated the diagnostic efficacy of liquid‐based cytology (LBC) in pancreatic juice cytology for pancreatic cancer.

**Methods:**

We retrospectively analyzed 24 patients with suspected pancreatic cancer that was difficult to diagnose by endoscopic ultrasound‐guided fine needle aspiration who underwent SPACE using LBC between April 2017 and April 2021.

**Results:**

The most common reason for performing SPACE was localized stenosis of the main pancreatic duct without a mass. Eleven patients were diagnosed with malignancy after surgical resection, nine of whom had pancreatic ductal adenocarcinoma. Ten patients were diagnosed as benign after a follow‐up of more than 1 year. The nine cases of malignancy were diagnosed before surgical resection by SPACE using LBC, with a sensitivity of 81.8% and specificity of 100%. The overall diagnostic accuracy was 91.7%. A total of 152 LBC examinations were performed via SPACE, with an adequate sample collection rate of 88.9%. No adverse events, including acute pancreatitis, occurred after endoscopic retrograde cholangiopancreatography.

**Conclusion:**

SPACE with LBC offers good diagnostic efficacy in patients with pancreatic cancer that is difficult to diagnose by endoscopic ultrasound‐guided fine needle aspiration.

## INTRODUCTION

The efficacy of endoscopic ultrasound‐guided fine needle aspiration (EUS‐FNA) in the diagnosis of pancreatic cancer is well‐established.^1^, ^2^ However, adverse events, such as needle tract seeding (NTS), have been reported.^3^, ^4^ In addition, its diagnostic accuracy for small lesions is low in the early stages.^5^, ^6^ Although carcinoma in situ (CIS) of the pancreas without mass formation occurs, its diagnosis by EUS‐FNA is not possible; instead, conventional diagnostic endoscopic retrograde cholangiopancreatography (ERCP) is required.^7^, ^8^ The efficacy of serial pancreatic juice aspiration cytological examination (SPACE) via an endoscopic naso‐pancreatic drainage (ENPD) tube has also been reported,^8^ but it is sometimes difficult to collect an adequate volume for analysis. The diagnostic performance of ERCP‐based tissue sampling may be inferior to that of EUS‐FNA,^9^ and the incidence of post‐ERCP pancreatitis (PEP) cannot be negligible.^10^, ^11^

On the other hand, the diagnostic efficacy of liquid‐based cytology (LBC) in EUS‐FNA has been reported.^12^, ^13^, ^14^, ^15^ However, no reports have evaluated LBC with SPACE. Hence, we examined the diagnostic efficacy of LBC for pancreatic cancer that is difficult to diagnose by EUS‐FNA.

## PATIENTS AND METHODS

### Study population

From April 2017 to April 2021, a total of 24 patients suspected of having pancreatic cancer underwent ERCP at Nara Medical University Hospital. All patients underwent imaging studies, including contrast‐enhanced computed tomography (CT) and magnetic resonance cholangiopancreatography (MRCP). EUS was performed prior to ERCP, and EUS‐FNA was also performed for cases with obvious pancreatic masses.

Our inclusion criteria were as follows: (1) patients with main pancreatic duct (MPD) stenosis in whom CT/MRCP/EUS could not confirm the presence of a mass, (2) patients with a pancreatic mass confirmed via EUS but for whom EUS‐FNA could not provide a definitive pathological diagnosis, and (3) patients with a pancreatic mass confirmed via CT/MRCP/EUS that was suggestive of an intraductal tumor, such as an intraductal papillary mucinous neoplasm (IPMN), and EUS‐FNA was avoided because of NTS concerns. Patients who refused to participate were excluded from the study.

This retrospective study was approved by our ethics committee (#3347). This study was performed according to the Helsinki Declaration of the World Medical Association. Written informed consent was obtained from all patients before performing ERCP. Owing to the retrospective nature of this study, an opt‐out approach on the website was used instead of requiring written informed consent for inclusion in this study.

### Endoscopic retrograde cholangiopancreatography

ERCP was performed while the patients were in a prone or semi‐prone position under conscious sedation using intravenous midazolam and buprenorphine hydrochloride or dexmedetomidine with CO_2_ insufflation. Pancreatography was carried out using a duodenoscope (TJF260V or JF260V; Olympus, Tokyo, Japan), and pancreatic duct cannulation was performed using a tapered catheter (ERCP CATHETER; MTW Endoskopie, Düsseldorf, Germany) with a 0.025‐inch guidewire (VisiGlide2; Olympus). SPACE was performed using a 5‐Fr ENPD tube (Nasal Pancreatic Drainage Set; Cook Medical, Japan). In patients with an ENPD, SPACE via the ENPD tube was repeatedly performed. Mikata et al. reported that performing pancreatic juice cytology (PJC) via ENPD six times is reasonable,^16^ and Iiboshi et al. reported good diagnostic accuracy of SPACE with an average of 5.3 PJC examinations.^8^ Based on these previous reports, we performed PJC examinations at least six times over 3–4 days.

### LBC samples

**FIGURE 1:**
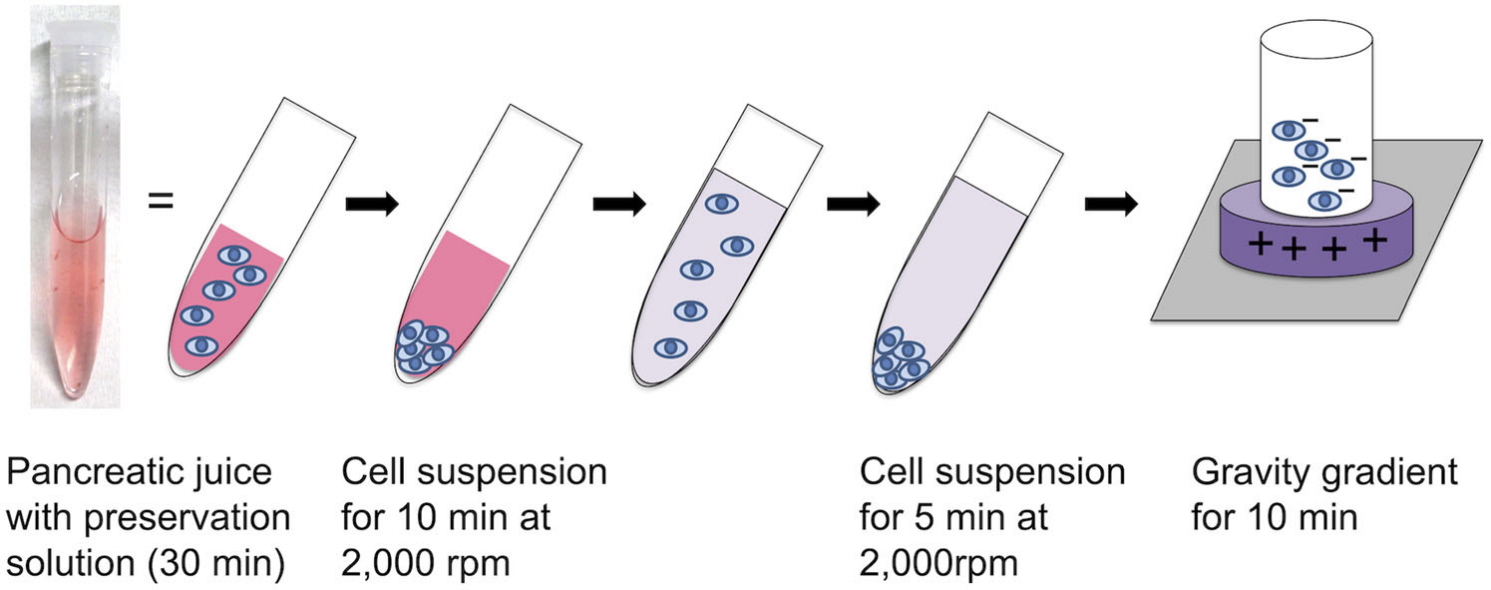
A schematic illustration of liquid‐based cytology. The collected pancreatic juice via an endoscopic naso‐pancreatic drainage tube should be immersed in the preservation solution for at least 1/2 h. The supernatant was discarded, and distilled water (DW) was added. The solution was centrifuged at 2000 rpm for 5 min. The supernatant was discarded, and 300 µl of DW was added. The solution was suspended and placed in a chamber attached to a glass slide that is positively charged in the center.

A small amount of PJ was collected in a sterilized tube from the ENPD tube, cooled with ice, and promptly brought to the cytology laboratory. The collected PJ was mixed with the prepared preservation liquid. The components of the preservative liquid were ammonium chloride, ammonium oxalate, ethanol, methanol, isopropanol, formalin, and ethylene glycol. This fixative lyses red blood cells and solubilizes proteins while preserving the diagnostically relevant materials. PJC specimens were assessed using LBC (SurePath method). Figure 1 shows the protocol for specimen treatment. Finally, we attached negatively charged cancer cells to the positively charged surface of the glass slide (BD SurePath PreCoat slides; Becton Dickinson, Japan).^17^ After washing the glass slide with distilled water, we obtained a homogenous and thin cell layer with a clear background on a limited area of the glass slide. The specimens (on glass slides) obtained from all patients were examined by two experienced pathologists. “Adequate samples” were defined as those that meet all criteria: 1) the pancreatic ductal epithelial cells have been collected in adequate numbers to be diagnostic; 2) the cell morphology was well preserved; and 3) the specimen preparation was good. Cytological diagnoses in adequate samples were classified into “normal or benign,” “indeterminate,” and “malignant or suspicious for malignancy.” “Malignant or suspicious for malignancy” was assumed as pathologically cancer‐positive. In the absence of consensus between the two pathologists, the final diagnosis was performed at the conference of the Department of Diagnostic Pathology.

### Follow‐up

All patients in the study underwent blood testing and physical examination on the day after the procedure to evaluate the occurrence of adverse events. The diagnosis and severity of all adverse events, including PEP, were based on the ASGE lexicon.^18^ After discharge, all patients with pathologically confirmed pancreatic cancer underwent surgical resection, and those with no evidence of malignancy on SPACE underwent periodic outpatient imaging tests after discharge. The median follow‐up period was 25.3 months (interquartile range [IQR] 17.4–41.5 months).

### Endpoints and definitions

The primary endpoint was the diagnostic accuracy of SPACE using LBC for pancreatic cancer. The secondary endpoints were sample adequacy of the LBC method and post‐ERCP adverse events. Patients’ final diagnoses were confirmed through surgical resection of specimens or clinical follow‐up. The diagnoses of the resected specimens were based on the guidelines of the National Comprehensive Cancer Network.^19^ Patients who were consistently healthy, with pancreas without changes in the appearance of the MPD, and absence of tumor progression during the 1‐year follow‐up period were diagnosed with benign MPD stenosis. The median follow‐up period for nonresected cases was 23.2 months (IQR 16.7–31.7 months).

## RESULTS

### Patient characteristics

Table 1 shows the 24 patients’ characteristics. The median age was 73.5 years old (IQR 66.8–76.0 years old). The lesions were more commonly located in the pancreatic body/tail. The most common indication for SPACE was MPD stenosis with no mass formation as confirmed by imaging studies, followed by a small mass that was difficult to diagnose by EUS‐FNA and an intraductal tumor. Cases of intraductal tumors were difficult to directly biopsy using forceps because the lesion was either located in the pancreatic tail or the MPD of the pancreatic head was torturous. The final diagnosis was made based on the resected specimens, which were classified as pancreatic ductal adenocarcinoma (PDAC), IPMN/IPMC, or intraductal tubulopapillary neoplasm (ITPN). All IPMN cases were a mixed type of MPD and branch duct and formed masses in the MPD. Meanwhile, one patient with autoimmune pancreatitis underwent surgical resection for suspicion of pancreatic cancer on imaging studies despite negative SPACE results. All nonresected benign cases were diagnosed based on the clinical course during the follow‐up period of more than one year. The median follow‐up period for nonresected cases was 23.2 months (IQR 16.7–31.7 months).

**TABLE 1:** Patient characteristics

	**All patients (*n* = 24)**
Age (IQR), years	73.5 (66.8–76.0)
Sex (male/female), *n*	15/9
Location of the lesion (Ph/Pbt)	10/14
Reasons for SPACE	
Main pancreatic duct stenosis without mass confirmed by imaging studies, *n* (%)	16 (66.7)
EUS‐FNA did not provide a definitive diagnosis, *n* (%)	4 (16.7)
Intraductal tumor, *n* (%)	4 (16.7)
*Final diagnosis*	
*Malignant cases, n* = 11	
Pancreatic ductal adenocarcinoma, *n* (%)	9 (37.5)
Invasive carcinoma, *n* (%)	7 (29.2)
Carcinoma in situ, *n* (%)	2 (8.3)
Noninvasive IPMC (mixed type), *n* (%)	1 (4.2)
Invasive ITPN, *n* (%)	1 (4.2)
*Benign cases, n* = 13^＊^	
Low‐grade IPMN (mixed type), *n* (%)	1 (4.2)
Low‐grade PanIN, *n* (%)	1 (4.2)
AIP, *n* (%)	2 (8.3)
Benign pancreatic duct stenosis, *n* (%)	9 (37.5)
Follow‐up period (IQR), months	
Overall	25.3 (17.4–41.5)
Nonresected cases	23.2 (16.7–31.7)

Abbreviations: AIP, autoimmune pancreatitis; EUS‐FNA, endoscopic ultrasound‐guided fine needle aspiration; IPMC, intraductal papillary mucinous carcinoma; IPMN, intraductal papillary mucinous neoplasm; IQR, interquartile range; ITPN, intraductal tubulopapillary neoplasm; PanIN, pancreatic intraepithelial neoplasia; Pbt, pancreatic body or tail; Ph, pancreatic head; SPACE, serial pancreatic juice aspiration cytological examination.

*Low‐grade IPMN, low‐grade PanIN, and one case of AIP were diagnosed on the basis of the examination of resected specimens. The remaining 10 cases were diagnosed on the basis of their clinical courses.

### Serial pancreatic juice aspiration cytological examination

In 21 out of 24 cases, the ENPD tube could be inserted beyond the MPD stenosis and into the pancreatic tail. In three patients, the catheter did not pass through the MPD stenosis; instead, the tip of the ENPD was positioned proximal to the pancreatic head. After tube placement, a median of six PJC examinations was performed via ENPD in each patient. In total, 152 cytological examinations were performed, which was equivalent to an adequate sample collection rate of 88.9% (Table 2). The median serum amylase level of patients one day after ERCP was 231 IU/ml, and no patients developed PEP.

**TABLE 2:** Serial pancreatic juice aspiration cytological examination (SPACE) with liquid‐based cytology (LBC)

Numbers of cytology via ENPD in each patient, median, *n* (range)	6 (5–10)
Numbers of cytology via ENPD in all patients	152
Collection of adequate samples, *n* (%)	135 (88.9)
Serum amylase levels after ERCP, median (IU/ml), (IQR)	231 (123–559)

Abbreviations: ENPD, endoscopic naso‐pancreatic drainage; ERCP, endoscopic retrograde cholangiopancreatography; IQR, interquartile range; LBC, liquid‐based cytology; SPACE, serial pancreatic juice aspiration cytological examination.

### Diagnostic yield of LBC in SPACE for pancreatic cancer

The diagnostic performance for detecting malignancy is shown in Table 3. The diagnostic sensitivity, specificity, and accuracy were 81.8%, 100%, and 91.7%, respectively.

**TABLE 3:** Diagnostic ability of liquid‐based cytology (LBC)

	**Sensitivity (%)**	**Specificity (%)**	**PPV (%)**	**NPV (%)**	**Accuracy (%)**
LBC	81.8 (9/11)	100 (13/13)	100 (9/9)	86.7 (13/15)	91.7 (22/24)

Abbreviations: LBC, liquid‐based cytology; NPV, negative predictive value; PPV, positive predictive value.

### Malignant cases

**FIGURE 2:**
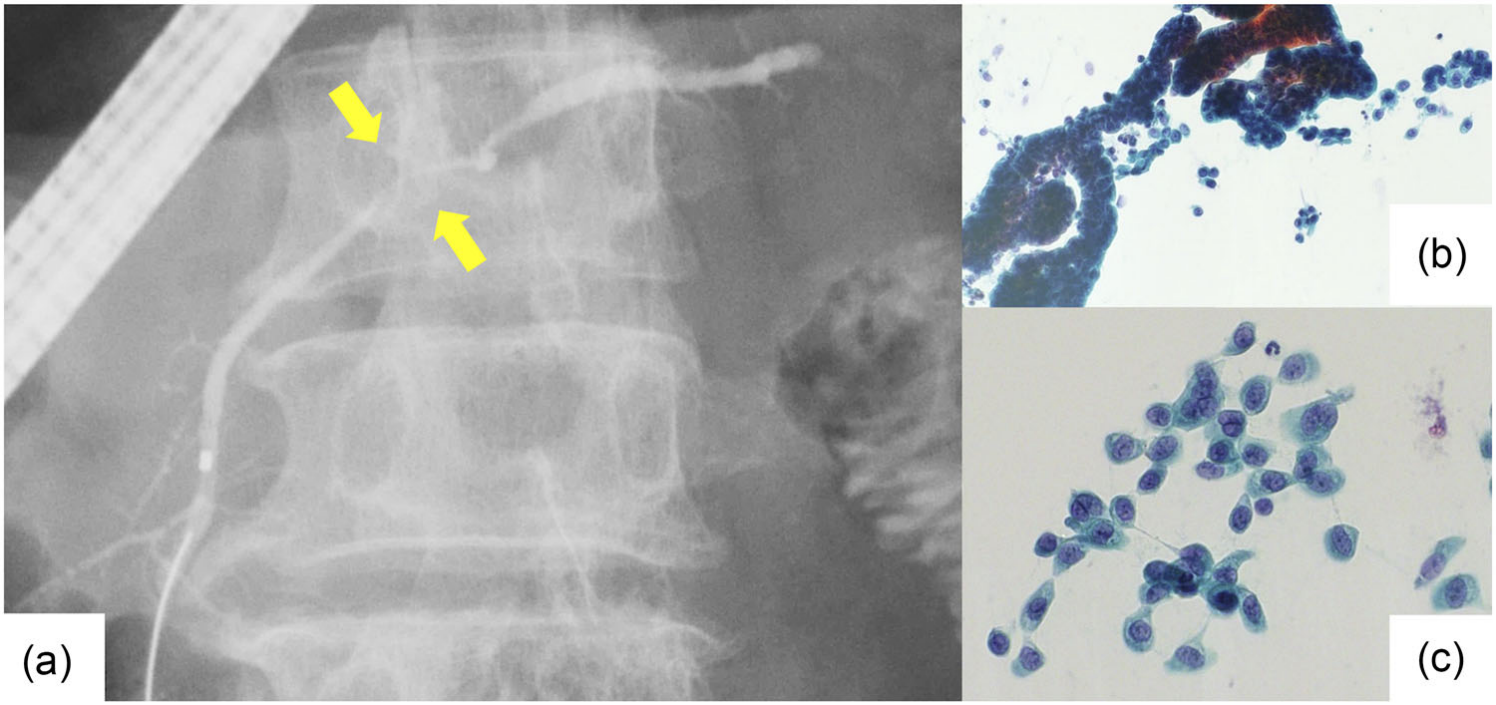
Findings of stage 0 pancreatic ductal adenocarcinoma (Case 5 in Table 4, carcinoma in situ). (a) Endoscopic retrograde cholangiopancreatography. Pancreatography revealed stenosis of the main pancreatic duct in the body of the pancreas. The patient had no mass detected in other imaging studies. An endoscopic naso‐pancreatic drainage tube was placed, and serial pancreatic juice aspiration cytology was performed. (b, c) Images of liquid‐based cytology. The background of inflammatory cells and artifacts is removed, and solitary, scattered tumor cells can be evaluated.

Malignancy was diagnosed in eleven patients (Table 4); all underwent curative surgical resection. The most frequent malignancy was PDAC (nine cases); two cases were stage 0 (CIS, Figure 2), two cases were IA, one case was IIA, and three cases were IIB. One patient underwent surgical resection after neoadjuvant chemoradiation therapy with no residual cancer cells in the resected specimen (Case 3, pathologically complete response). Additionally, one case each of invasive ITPN and noninvasive IPMC was also observed. Of these eleven cases, nine were diagnosed as cancer‐positive by SPACE before surgical resection. However, two cases were not diagnosed by SPACE (Cases 3 and 8). Case 3 was diagnosed as adenocarcinoma by pancreatic ductal brush cytology performed simultaneously during ERCP. In Case 8, a pancreatic tumor that was not initially identified became apparent 3 months later and was diagnosed as adenocarcinoma by EUS‐FNA, with surgical resection being subsequently performed.

**TABLE 4:** Malignant cases

**Case**	**Age (years)**	**Sex**	**Location**	**Final diagnosis**	**LBC diagnosis**	**Therapy**	**Stage**	**Follow‐up periods (months)**	**Status**	**Recurrence**
1	53	M	Pt	Invasive ITPN	Malignant (ca.)	Surgery + AC	IIA	50.0	Alive	Liver metastasis
2	76	F	Ph	Noninvasive IPMC	Malignant (adenoca.)	Surgery	0	51.8	Alive	No
3	72	M	Pt	PDAC	Indeterminate	NACRT + Surgery + AC	pCR	13.2	Alive	No
4	75	F	Pb	PDAC	Malignant (adenoca.)	Surgery + AC	IIA	13.5	Alive	No
5	66	M	Pb	PDAC	Malignant (adenoca.)	Surgery + AC	0 (CIS)	52.6	Alive	No
6	67	F	Ph	PDAC	Malignant (adenoca.)	Surgery + AC	0 (CIS)	19.1	Alive	No
7	72	F	Ph	PDAC	Malignant (adenoca.)	NACRT + Surgery + AC	IA	40.6	Alive	Peritoneal dissemination
8	73	M	Ph	PDAC	Indeterminate	Surgery + AC	IIB	49.9	Alive	No
9	76	F	Ph	PDAC	Malignant (adenoca.)	Surgery + AC	IIB	34.1	Alive	Lung metastasis
10	76	M	Pt	PDAC	Malignant (adenoca.)	NACRT + Surgery + AC	IA	22.5	Death	Lung metastasis, liver metastasis
11	76	M	Pt	PDAC	Malignant (adenoca.)	NACRT + Surgery + AC	IIB	24.0	Alive	No

Abbreviations: AC, adjuvant chemotherapy; adenoca., adenocarcinoma; ca., carcinoma; IPMC, intraductal papillary mucinous carcinoma; IPMN, intraductal papillary mucinous neoplasm; ITPN, intraductal tubulopapillary neoplasm; LBC, liquid‐based cytology; NACRT, neoadjuvant chemoradiation therapy; Pb, pancreatic body; pCR, pathologically complete response after NACRT; PDAC, pancreatic ductal adenocarcinoma; Ph, pancreatic head; Pt, pancreatic tail.

## DISCUSSION

PDAC is the most common type of pancreatic cancer and has a very high mortality rate.^20^ The reasons for the difficulty of early diagnosis and therapy of pancreatic cancer include the absence of early‐stage biomarkers, anatomical location of the retroperitoneum that allows invasion of surrounding organs and blood vessels, and nonspecific symptoms. In recent years, the number of pancreatic cancer cases that were detected at an early stage has been increasing, and in these cases, MPD stenosis or dilatation is often noted even if the pancreatic tumor cannot be detected by CT or MRCP. It is increasingly recognized that the prognosis for patients with early‐stage pancreatic cancer is favorable. According to the Japanese Pancreatic Cancer Registry, the 5‐year survival rates for patients with stage 0 (CIS), stage IA, and stage IB cancers were relatively good.^21^ However, in cases of small pancreatic tumors, EUS often fails to delineate the tumor, and FNA is not feasible. Furthermore, even if needle puncture by EUS‐FNA is successful, there is a possibility of NTS after surgical resection.^3^, ^4^ Thus, a clinical dilemma arises: the earlier clinicians try to accurately diagnose pancreatic cancer, the more difficult to diagnose the disease by EUS‐FNA becomes.

EUS‐FNA is the first diagnostic option for pancreatic solid tumors and has high diagnostic accuracy and safety.^1^, ^2^ In recent years, needles have been specifically designed for collecting sufficient tissue specimens. The overall sensitivity of EUS‐FNA for the diagnosis of pancreatic cancer is approximately 90%; however, its diagnostic ability for small lesions remains limited.^5^, ^6^ EUS‐FNA is not indicated for cases in which a mass cannot be delineated by EUS. Specifically, this applies to cases of noninvasive cancer (CIS) that do not form a mass and to cases in which a pancreatic mass cannot be visualized by conventional B‐mode EUS. In these cases, ERCP‐based tissue sampling is the first‐line diagnostic method.

Cytological diagnosis using PJ collected via ERCP has several problems. Usually, PJ collected by ERCP is cytologically examined by the smear method as it is simple and inexpensive. However, the amount of cells placed on the glass slide depends on the skill of the operator, and dry denaturation results in poor cell preservation. Although PJC via ERCP has been performed since the introduction of ERCP, the diagnostic sensitivity for pancreatic cancer varies between reports, ranging from approximately 30% to 90%.^22^, ^23^, ^24^, ^25^, ^26^ On the other hand, Mikata et al. and Iiboshi et al. reported SPACE for cytological diagnosis by collecting PJ multiple times via an ENPD.^8^, ^16^ This method may increase the diagnostic sensitivity when compared to collecting PJ during ERCP and submitting it for a single cytological examination. However, reports on the diagnostic accuracy of SPACE are scarce, and there is insufficient evidence to support its diagnostic efficacy. Issues, such as specimen treatment or operator skill, may also cause disparities in diagnostic accuracy among facilities.

LBC, which was developed in the 1990s, began with cervical cytology and has since been widely applied to various organs.^27^, ^28^, ^29^ LBC has the following advantages: first, cells can be efficiently transferred from the collection device, and almost all cells can be analyzed. Second, the use of separation reagents can selectively eliminate red blood cells, inflammatory cells, and mucus, thus focusing the analysis on cells necessary for diagnosis and reducing inadequate smears. Third, the remaining cytoplasm can be used for immunostaining and genetic testing. In particular, the first and second advantages, that is, the high efficiency of cell collection and the ability to selectively attach tumor cells to glass slides may have contributed to the good diagnostic accuracy of LBC in the current study. On the other hand, the disadvantages of LBC include the labor‐intensive and costly preparation of cytology specimens. Additionally, treatment with LBC may result in morphological changes and the destruction of architectural features of cells. In recent years, the diagnostic efficacy of LBC in EUS‐FNA for pancreatic tumors and in PJC of IPMN has been reported.^12^, ^13^, ^14^, ^15^, ^30^ The LBC method may be able to minimize the variations in diagnostic accuracy between institutions, which is mainly caused by specimen treatment. Therefore, in our study, we decided to use the LBC method for specimen treatment to improve the diagnostic performance of SPACE in pancreatic tumors that are difficult to diagnose by EUS‐FNA. In the current study, the diagnostic accuracy for malignancy was 91.7%. Additionally, the collection rate of adequate samples was 88.9%. In 2022, Kawamura et al. reported that in ERCP‐based cytology in the preoperative pathological diagnosis of PDAC,^25^ the sensitivity of PJC via ENPD was only 30.8%. In their study, the rate of adequate samples collected from ENPD was low (69.5%), which may have reduced diagnostic sensitivity. The LBC method has the potential to improve the diagnostic accuracy of PJC by increasing the rate of adequate sample collection in SPACE.

In our study, no cases of PEP occurred. We tried to place the ENPD tube as deeply as possible beyond the stenosis of the MPD, and carefully monitored the amount of pancreatic juice draining from the ENPD after ERCP. This may have contributed to the safety of the ERCP procedure, as the pancreatic juice was efficiently drained and the intraductal pancreatic pressure did not increase.

In the current study, there were two false‐negative malignant cases that could be not diagnosed as cancer‐positive by SPACE using the LBC method. In one of these cases, PDAC was confirmed by brush cytology of the pancreatic duct performed simultaneously during ERCP. It may be difficult to insert a cytology brush catheter in cases wherein the lesion is in the tail of the pancreas or the pancreatic duct is tortuous. However, in cases where catheter insertion is easy, it would be desirable to perform pancreatic duct brush cytology simultaneously with ERCP to complement the diagnostic capabilities of SPACE. In the other false‐negative case, the pancreatic tumor was not observed at the time of SPACE, and the only finding was MPD stenosis. However, during the follow‐up period, the pancreatic mass was observed, and the diagnosis of PDAC was made by EUS‐FNA. Therefore, even in cases malignancy was not detected by SPACE, close follow‐up is necessary as there is the possibility of false‐negative results. Meanwhile, no false‐positive cases were observed in the current study.

Our study has several limitations. The first is that we used the LBC method throughout SPACE; thus, a comparison with the classical smear method was not performed. Future comparison studies between the classical smear method and the LBC method may be necessary. Second, this was a retrospective study of a relatively small number of cases at a single center. In fact, many pancreatic tumors can be diagnosed using EUS‐FNA, and SPACE is indicated in fewer cases than EUS‐FNA. Therefore, a prospective multicenter study with a larger number of cases may be needed in the future.

## CONCLUSION

The findings of the current study suggest that SPACE with LBC offers good diagnostic efficacy in patients with pancreatic neoplasms that are difficult to diagnose by EUS‐FNA.

## CONFLICT OF INTEREST

The authors declare that they have no conflict of interest.

## FUNDING INFORMATION

None.

## Data Availability

The datasets used and/or analyzed during the current study are available from the corresponding author upon reasonable request.
